# Rationale, design, and methodology of My Body Is Fit and Fabulous at school (MyBFF@school) study: a multi-pronged intervention program to combat obesity among Malaysian schoolchildren

**DOI:** 10.1186/s12889-024-20726-z

**Published:** 2025-01-10

**Authors:** Abdul Halim Mokhtar, Ruziana Mona Wan Mohd Zin, Abqariyah Yahya, Fuziah Md. Zain, Rusidah Selamat, Zahari Ishak, Muhammad Yazid Jalaludin

**Affiliations:** 1 Department of Sports Medicine, Faculty of Medicine, Universiti Malaya, Kuala Lumpur, Wilayah Persekutuan Kuala Lumpur 50603 Malaysia; 2 Faculty of Sports and Exercise Science, Universiti Malaya, Kuala Lumpur, 50603 Malaysia; 3 Department of Pediatrics, Faculty of Medicine, Universiti Malaya, Kuala Lumpur, Wilayah Persekutuan Kuala Lumpur 50603 Malaysia; 4 Endocrine and Metabolic Unit, Nutrition, Metabolic & Cardiovascular Research Centre, Institute for Medical Research, National Institute of Health (NIH), Ministry of Health, Setia Alam, Shah Alam, Selangor 40170 Malaysia; 5 Department of Social and Preventive Medicine, Faculty of Medicine, Universiti Malaya, Kuala Lumpur, Wilayah Persekutuan Kuala Lumpur 50603 Malaysia; 6 Department of Pediatrics, Putrajaya Hospital, Ministry of Health, Jalan P9, Pusat Pentadbiran Kerajaan Persekutuan Presint 7, Putrajaya, Wilayah Persekutuan Putrajaya 62250 Malaysia; 7 Nutrition Division, Ministry of Health Malaysia, Federal Government Administrative Centre, Level 1, Block E3, Complex E, Putrajaya, Wilayah Persekutuan Putrajaya 62590 Malaysia; 8 FOSSLA, UCSI University, Kuala Lumpur, Wilayah Persekutuan Kuala Lumpur 56000 Malaysia

**Keywords:** Overweight, Obesity, Primary School, Secondary School, Intervention

## Abstract

**Background:**

Childhood obesity has increased rapidly in recent years and is now a global epidemic. To combat this, MyBFF@school program, a multi-faceted obesity intervention incorporating physical activity in the form of small-sided games (SSG), nutrition, and psychology components for schoolchildren was designed. This paper is aimed at describing the protocol of the MyBFF@school program and presenting the baseline findings including the overweight and obesity prevalence.

**Methods:**

MyBFF@school is a school-based, cluster randomized controlled trial (C-RCT) study. The investigators selected government schools from Federal Territory of Kuala Lumpur, Selangor and Negeri Sembilan by stratified proportionate random sampling based on the multi-ethnic population and the urban–rural location of schools. Subsequently, the schools were assigned randomly to intervention and control groups. The intervention schools underwent MyBFF@school program, whereas the control followed standard school curriculum for a duration of six months. The intervention modules replaced the existing two physical education classes and one co-curriculum activity per week. Three assessments i.e. at baseline, month-3 and month-6 were conducted. Anthropometric, clinical examination, blood, physical fitness, nutrition, and psychology parameters were collected.

**Results:**

Twenty-three out of 1,196 primary schools (seven interventions and 16 controls) and 15 out of 416 secondary schools (six interventions and nine controls). The investigators screened 11,950 primary (age 9–11 years) and 10,866 secondary (age 13, 14, 16 years) schoolchildren. The investigators found 3,516 primary schoolchildren (29.4%) and 2,910 secondary schoolchildren (26.8%) had BMI *z*-score of more than + 1SD who were eligible for the study. Of these, 39.7% (N = 1397) of the primary and 35.8% (N = 1041) of the secondary schoolchildren agreed to participate in the study. The mean (SD) characteristics for the participating primary and secondary schoolchildren were: BMI *z*-score, + 2.29 (± 0.81) and + 2.10 (± 0.71); waist circumference, 75.06 (± 9.6) cm and 85.5 (± 10.9) cm; percentage body fat, 37.8% (± 6.5%) and 39.2% (± 7.3%); and muscle mass, 14.7 (± 2.9) and 23.1 (± 5.2) kg respectively.

**Conclusion:**

MyBFF@school program, a school-based multi-pronged intervention was designed to combat childhood obesity. Screening of 22,816 primary and secondary schoolchildren found 29.4% of primary schoolchildren and 26.8% of secondary schoolchildren to be overweight and obese which reflected the urgency for an effective intervention.

**Trial registration:**

Clinical trial number: NCT04155255, November 7, 2019 (Retrospective registered). National Medical Research Register: NMRR-13–439-16563. Registered July 23, 2013. The intervention program was approved by the Medical Research and Ethics Committee (MREC), Ministry of Health Malaysia and Educational Planning and Research Division (EPRD), Ministry of Education Malaysia. It was funded by the Ministry of Health Malaysia.

## Background

Childhood obesity has become a global epidemic. Both the prevalence and severity of obesity have increased in recent years, likely as a result of complex interactions between genes, dietary intake, physical activity, social and the environment [1–4]. However, meta-analysis and a review of reviews found that school-based childhood obesity intervention normally incorporated only physical activity and nutrition aspects [5, 6]. According to the International Obesity Task Force, the global prevalence of overweight and obese children and young adults aged between 5 and 17 years is approximately 10%, of which 2%–3% were obese [7]. Furthermore, reports have shown that pediatric obesity epidemic has spread globally, particularly to countries undergoing economic transition [8].

Malaysia is one of many countries dramatically affected by this growing epidemic. The National Health and Morbidity Survey (NHMS) found a consistently increasing trend of obesity among Malaysian adults from 2006 to 2015 [9]. This trend was also observed among children under the age of 17 with the prevalence of obesity showed increment from 11.9% in 2015 to 14.8% in 2019 [10]. In addition, the Nutrition Survey of Malaysian Children has reported high prevalence rates of overweight and obese children aged 6–12 years: 14.4% and 20%, respectively [11].

Obesity poses many serious health and financial problems. Excess body weight during adolescence can indicate the risk of adult obesity [12], and it also influences morbidity and mortality in adult life. Childhood obesity increases the risk of premature mortality and is associated with a significantly increased risk of cardiometabolic morbidity, such as diabetes, hypertension, heart disease, and strokes in adult life [13, 14]. In addition, childhood obesity influences quality of life, having impacts on physical, social, and psychological functioning [15]. Hence, the obesity epidemic demands an urgent childhood obesity intervention program to prevent and curb this problem. Treating childhood obesity is often time-consuming, difficult, expensive [16] and rarely results in significant and sustainable weight loss necessary to improve health outcomes. Recent clinical guidelines have recommended healthy eating, increased physical activity, and a supportive environment as the primary goals for treating and managing pediatric obesity [17].

Schools are popular settings for promoting lifelong healthy eating and physical activity, and thus for preventing and combating childhood obesity. Most strategies to address childhood obesity focus on school-based intervention. Several systematic reviews and studies have shown that school-based prevention of obesity is both feasible and effective [18–21]. For example, the “Happy 10 program,” developed as a classroom-based intervention to promote physical activity in Chinese primary school students, was shown to be feasible, well-accepted, and effective in reducing weight gain [22], and its significant effects were sustained even one year after the program ended [23]. Another such program, “Lekker Fit! (Enjoy being fit!),” was conducted in primary schools in the Netherlands and focused on promoting healthy eating and an active lifestyle. The program was shown to be effective in reducing weight and waist circumference, as well as improving the fitness level of the participating overweight children [24]. Despite the effectiveness of school-based intervention found in a number of studies, not all such programs are successful. A school-based program in Birmingham, United Kingdom, for example, did not result in the predicted improvement [25].

When designing an intervention program, ample consideration should be applied on the types of intervention and its feasibility. An article that looked critically into childhood obesity school-based intervention highlighted that major obstacles to obesity interventions include schools lacking resources and infrastructure to implement the intervention program [26]. The intervention proposed should be feasible using the available resources of the schools. This includes infrastructure, personnel, and equipment required for implementation, to name a few besides the policy and administration that support the intervention.

Type of school-based obesity interventions ought to be multi-component to be successful. A recent meta-analysis supported multi-component intervention (diet and physical activity) and modification of Physical Education (PE) curriculum (increase in exercises or physical activities) to improve obesity outcomes (adiposity and physical activity) [27]. This is accorded by another meta-analysis on effectiveness of school-based interventions in mainland China. The authors included 76 studies (randomised and non-randomised control trials) which involved 72,620 schoolchildren aged 6 to 19 years old and found that comprehensive interventions with physical activity and health education had larger effect on change of BMI than physical activity only interventions [28]. Khambalia et al. reviewed three meta-analysis and five systematic reviews between the years 1990–2010 and synthesised that schoolchildren obesity intervention that resulted in significant weight reduction included long-term interventions with combined diet and physical activity and a family component. They also concluded that limited evidence was found on which to base recommendations including no single intervention will fit all schools and populations [29].

WHO guidelines on physical activity and sedentary behaviour (2020) recommended children and adolescents aged 5–17 years to do at least an average of 60 min per day of moderate-to-vigorous intensity, mostly aerobic, physical activity a week as they are associated with improved health outcomes [30]. Including physical activity (PA) component in a school-based obesity intervention would be able to promote this. A recent systematic review and meta-analysis concluded that interventions with a PA component in schoolchildren with obesity seem to be successful in reducing BMI and produce an increase in time spent engaged in PA, especially moderate-to-vigorous activity (MVPA). Therefore, interventions based on PA should be considered one of the main strategies to be implemented to fight childhood obesity [31]. In a meta-analysis by Hong Mei et al., one of the findings was an inconsistent impact of PA on obesity across 18 studies around the globe (USA, Europe, Asia and Africa). The population studied was 22,381 primary schoolchildren aged 6–12 years. Nevertheless, the authors did conclude that long term PA intervention had a significant impact on reducing BMI gain [5]. An interesting finding by the same study was that shorter PA time was better. The authors found that a PA intervention of < 100 min/week was better than ≥ 100 min/week and suggested the importance of quality rather than quantity of the PA intervention. There are other reviews that supported higher intensity, longer duration of MVPA and variety of activities in school-cased PA intervention to be more effective [32, 33].

A survey involving 1718 overweight and obese adolescents in the 9th–12th grade (majority 9th and 10th) i.e. between the age of 14–18 years old and their parents found that among various types of physical activity interventions for adolescents, sports participation proved to be among the most effective. The other physical activities included in the survey for comparison included extra-curricular physical activity, active commuting, physical education, and recreational activity for fun [34]. Nevertheless, this opinion is not shared by all. A systematic review included studies directly compared sport participants with those non-participants weight status, physical, and diet. The authors found no clear association between sport participation and body weight, but sports participants were found to be more physically active. Diet outcomes provided mixed results with increased fruit, vegetable, and milk consumption, yet an increase in fast food and sugar sweetened beverages and greater overall calorie intake too [35]. A cross-sectional study comparing team versus individual sports participation revealed more advantage with team sports. The researchers studied 756 children and adolescent athletes between the ages of 6 to 18 years. They found 8% of the population studied reported anxiety or depression, with the individual sports athletes reported more (13% versus 7%, *p* < 0.01). In addition, team sports athletes play more for fun compared to individual sports athletes who play more for goal-oriented reasons (e.g. for scholarship) [36]. On top of this team sports can increase participation through enjoyment, social interaction, and bonding [37]. A Danish study on team sports using SSG in 300 schoolchildren aged 8–10 years old and compared to individual sports intervention (circuit strength training and interval running), found that enjoyment and social cohesion did not reduce in team sports, but reduced significantly in individual sports. The authors concluded that team sports seem to be more advantageous for the development of enjoyment and cohesion [38].

Types of exercise could contribute to different magnitudes of weight loss. A review mainly on adult subjects found that aerobic exercise interventions for 6–12 months without caloric restriction yield a 2–3% weight loss and a dose–response relationship exists between aerobic intensity and weight loss. Resistance training without caloric restriction improved body composition but might not yield significant weight loss. When combined with caloric restriction, resistance training could induce weight loss [39].

The school curriculum incorporated physical activity interventions through obligations of PE periods [40, 41], physical activity homework [40], before and after-school activities [42, 43]. Unfortunately, the PE period may not involve sufficient intensity and duration to burn out the excessive calorie intake [44]. A study in Malaysian schools published in 2012 found that the physical education program was not adequately implemented and was contributed by shortage of sports facilities, lack of opportunities for the children to be active, status of the PE inside schools and lack of qualified PE teachers [45]. More attention should be given to PE classes as they are the main physical activity intervention at school.

Summarizing the literature on physical activity intervention for children and adolescent obesity, the team of investigators outline that an effective obesity intervention program should include physical activity component, and that the physical activity should be of moderate-to-vigorous, moderate to high intensity, aerobic rather than resistance training, sports oriented rather than non-sports exercise type and team sports rather than individual sports. On top of that, the implementation should maximize the school physical education classes and resources available at school.

School health education has an impact on childhood obesity [46]. The advantage of school health education is that it aims to instil awareness in schoolchildren of healthy lifestyles and dietary habits [47] and this can prevent obesity [48]. Nutrition component of the health education ought to improve students' food literacy, equip them with food preparation skills and enhance physical activities [49]. An effective nutrition education is considered when nutrition knowledge improvement accompanied by positive change in dietary practice [48]. Several key points were highlighted by a systematic review on effectiveness of nutrition education on obese children and adolescents which included introduction of nutrition education into the regular curriculum and duration of more than a year [50].

Obese children are known to have several psychological issues [51]. Many of them are unhappy with their body size and shape, and they face teasing and jeering from family members and school mates [52]. They may have low self-esteem and self-worth [53]. At school, they may be bullied, made fun of for their fatness and given nicknames by their schoolmates [54]. At times, they may bully others possibly to subconsciously overcome their own inferiority feelings owing to obesity and body image dissatisfaction [55].

Stress, either acute or chronic, may affect obesity. During a stress episode, cortisol is secreted to improve appetite, especially sugar and fat [56]. This may lead to increased caloric intake and worsen the obesity. Chronic stress was shown to increase BMI among adolescents [57]. Furthermore, obese children are reported to suffer anxiety [58] and depression [59, 60]. The symptoms may be partial [61], and manifest as aggressive behaviour, anger and bullying [59, 60]. Some of them may end up eating more [62]. Anxiety and depression can be a cause and a consequence to childhood obesity [51], hence including a psychological intervention in obese child could be beneficial on both directions.

Physical activity could possibly mediate stress reduction and associated obesity. A cross-sectional study on fitness, stress and body composition in primary schoolchildren (average age 7.3 years old) found that high fitness was associated with less unfavourable body composition and elevated school stress [63]. Another study found that physically active girls could mitigate the BMI growth even when reporting high long-term stress, compared to less physically active girls. The study was a prospective study that followed up 2,379 school girls starting at the age of 9 or 10 year-old collecting 10 measurements over the span of 10 years; on BMI every year, and stress and physical activity every other year [64]. Another study on 303 boys aged 16.6 years found that PA appeared to buffer stress on adiposity [65].

In essence, the literature has impressed the burden of the obesity problem in children and rationalised the intervention to be school-based, multi-component, combining any two of these components: physical activity, nutrition and psychology. However, the outcome of interventions are not always successful and consistent. Physical activity and nutrition are shown to have synergistic effect and there is evidence that physical activity is beneficial to the psychology of obese schoolchildren. A combination of these three would be logical in tackling this issue.

The urgent need to address obesity in Malaysian schoolchildren led us to design the “My Body is Fit and Fabulous” (MyBFF@school) program, which is specifically targeted at overweight and obese schoolchildren. The MyBFF@school program is a multi-faceted obesity intervention strategy incorporating physical activity component in the form of small-sided games (SSG), together with nutrition and psychology education components. The SSG was hypothesized to increase physical activity within the relatively short period (less than 100 min/week) and meeting the moderate-to-vigorous intensity of physical activity. Meanwhile, the nutrition component was complemented with practical and interactive sessions to improve knowledge, practice and attitude. At the same time, psychology modules could help to reduce stress and improve adherence to the program. The requirements for the intervention package to be implemented in the future were considered to be within the reach of current school resources. For example, SSG would require a football field (commonly available at schools) adjusted to smaller size and football equipment. While, the nutrition and psychology modules would use the school classes and available space. Additional training to the current teachers would be required or an additional post to run the program could be the alternative choice during actual implementation. This paper is aimed at describing the protocol of the MyBFF@school program and presenting the baseline findings. The subsequent papers in this supplement will describe the other outcomes i.e. the body composition (Wan Mohd Zin RM, Mokhtar AH, Yahya A, Zain FM, Selamat R, Ishak Z, Jalaludin MY: Effects of MyBFF@school, a multifaceted obesity intervention program, on anthropometry and body composition of overweight and obese primary schoolchildren, unpublished), (Ahmad Kamil NZI, Mokhtar AH, Yahya A, Zain FM, Selamat R, Ishak Z, Jalaludin MY: Effects of MyBFF@school, a multifaceted obesity intervention program, on anthropometry and body composition of overweight and obese adolescent schoolchildren, unpublished), cardiometabolic markers (Jalaludin MY, Roslan FA, Mansor F, Zain FM, Hong JYH, Wan Mohd Zin RM, Ahmad Kamil NZI, Yahya A, Ishak Z, Selamat R, Mokhtar AH. Cardio-metabolic outcome of MyBFF@school intervention program among primary schoolchildren: a cluster randomized controlled trial, unpublished), physical activity (Mokhtar AH, Kamarudin MA, Choong A, Singh L, Genisan V, Yahya A, Wan Mohd Zin RM, Zain FM, Selamat R, Ishak Z, Jalaludin MY. The effect of the MyBFF@school program on cardiorespiratory fitness in overweight and obese primary schoolchildren: a cluster randomized controlled trial, unpublished), nutrition (Selamat R, Aziz NAA, Raib J, Zulkafly N, Mohamad WNAW, Ismail AN, Jalaludin MY, Zain FM, Ishak Z, Yahya A, Mokhtar AH. Effects of a nutrition education intervention on nutrition knowledge and attitude among overweight and obese primary schoolchildren: a cluster randomized controlled trial, unpublished), and psychology (Ishak Z, Fin LS, Wan Ibrahim WAH, Yahya A, Zain FM, Selamat R, Jalaludin MY, Mokhtar AH: Effects of MyBFF@school intervention in health-related quality of life among overweight and obese primary schoolchildren: a cluster randomized controlled trial, unpblished), (Ishak Z, Fin LS, Wan Ibrahim WAH, Yahya A, Zain FM, Selamat R, Jalaludin MY, Mokhtar AH: The effectiveness of MyBFF@school intervention program in reducing emotional and behavioral problems in overweight and obese secondary schoolchildren in Malaysia: a cluster randomized controlled trial, unpublished).

## Methods

### Study setting and sampling strategies

This school-based, cluster randomized controlled trial (C-RCT) study was primarily aimed at evaluating the effectiveness of the MyBFF@school program on overweight and obese schoolchildren’s BMI for age (BMI *z*-score) and percentage body fat (PBF) after three and six months of intervention. Prior to the commencement of the study, a complete list of public primary and secondary schools located in selected regions (Federal Territory of Kuala Lumpur, Selangor and Negeri Sembilan) was obtained from the Ministry of Education and used as the sampling frame. A total of 1,196 primary and 416 secondary public schools were identified. Proportionate random sampling was used to select schools that ensured sufficient representation of multi-ethnic populations in the study sample. These schools were stratified according to school type (either national or vernacular) and location (urban or rural). Urban schools were defined as schools located in the city with a population of not less than 10,000 people, whereas rural school are schools located in suburb a population not more than 10,000 people based on the Ministry of Education’s classification [66]. Following this, random sampling was used to randomly allocate schools to either the intervention or control arm of the study. Concealment of allocation was not possible because the list of selected schools must be submitted to the Ministry of Education for approval before beginning the study.

The selected intervention schools participated in the MyBFF@school program, whereas the control schools continued to follow the existing standard national school curriculum. The MyBFF@school intervention program was conducted by trained personnel who were stationed full-time at each intervention school for a duration of six months between February 2016 and August 2016. This study was approved by the Medical Research Ethics Committee (MREC), Ministry of Health, Malaysia (NMRR-13–439-16,563).

### Sample size estimation

A sample size estimation was established based on the main outcome parameter of mean difference in percentage body fat. The investigators begin by computing a sample size for standard RCT (individuals’ randomization) (N) where independence of samples is assumed. In this standard RCT, to achieve 80% power at 5% significance level, a minimal number required to detect a mean difference of 0.35 in percentage body fat was 804 (402 per arm) for each primary and secondary school. This is based on the investigators’ unpublished findings on changes of percentage body fat and attrition rate in a pilot study [67]. Assuming an intraclass correlation coefficient (ICC) of 0.01, cluster size of 50 and 50% attrition rate, the investigators decided to include 24 schools and a minimum of 1,200 subjects. In this study, the desired sample size was reached with 23 primary schools and 15 secondary schools.

### Participants’ eligibility

Inclusion criteria for the study were that schoolchildren should be overweight or obese, with a BMI for age of more than + 1 SD based on the WHO 2007 Growth Reference. Primary schoolchildren aged 9 to 11 years old (Year 3, 4, 5), while secondary schoolchildren aged 13, 14, and 16 years old (Form 1, 2 and 4). Thus, Year and Form refer to the number of years in the primary and secondary school respectively. Schoolchildren aged 12 (Year 6) and 15 (Form 3) were excluded from this program as they were involved in major national examinations, Primary School Achievement Test (UPSR) and Form Three Assessment (PT3) consecutively. Schoolchildren with a BMI for age below and/or equal to + 1 SD, with physical or mental disability, medical conditions that prevented their participation in moderate to vigorous physical activities, co-morbidities that may interfere with the study (such as diagnosed type 2 diabetes mellitus, hypertension, nephritic syndrome, epilepsy, congenital heart disease and skeletal anomalies), or a requirement for steroids, anti-epileptic treatments, or methylphenidate, were excluded from this study. These exclusion criteria were checked through self-reporting, clinical questionnaire and examination by medical doctors (pediatric and sports medicine).

All schoolchildren in the participating schools were screened, and those fulfilling the inclusion criteria were invited to participate in this study. A total of 11,950 and 10,866 schoolchildren were screened in the primary and secondary schools, respectively, of which 3,516 (29.4%) primary schoolchildren and 2,910 (26.8%) secondary schoolchildren were eligible for the study. Written informed consent and assent were obtained from both parent/guardian and schoolchildren.

### MyBFF@school intervention package

The intervention package comprised of physical activity, nutrition, and psychology components. All components were carried out during physical education periods and co-curriculum activities in the school during school hours and monitored by the investigators on a monthly basis, but formal assessment was performed at baseline, three and six months. The intervention was done in such a way for several reasons. Firstly, activities after school hours tend to yield a high drop-out rate as seen in the pilot study [67], (Mokhtar AH, Ishak Z, Zain FM, Selamat R, Yahya A, Jalaludin MY: An introduction to MyBFF@school, a school-based childhood obesity intervention program: a cluster randomized controlled trial, unpublished). Secondly, this program could not be added on the existing school curriculum as this would prolong the already packed school timetable and tight period (duration) of the curriculum. This was discussed between the investigators and stake-holders during the planning stage of MyBFF@school study. Finally, the feasibility of this intervention has been considered to be embedded (modified to suit) or to replace the existing physical education and co-curriculum activities in the future.

### Physical activity component

Physical activity component consisted of small-sided games (SSG) module. SSG sessions were conducted for 30 min for primary schools and 40 min for secondary schools as allocated in the school curriculum. Two sessions were organized per week; therefore, a total of 60 and 80 min per week were spent on SSG activities for primary school and secondary schoolchildren respectively. During the SSG session, participants were divided into teams of four to seven players on each side. The number of participants and the relatively small pitch allowed more play, touches, and runs. This would contribute to the intensity of the game as demonstrated by previous studies [68, 69]. It was expected to be moderate-to-vigorous or high intensity as reported by a study [70]. Another study conducted on 12 healthy untrained adolescent boys age 15.8 ± 0.6, found that SSG to be moderate-to-vigorous exercise intensity i.e. over 70% of peak heart rate [71]. The same study found no significant difference in perceived enjoyment in SSG compared to repeated sprint. The size of the pitch was approximately 14 m by 9 m, depending on the school’s available pitch. This size was similar to a volleyball court or one eighth of the standard football field. A study on the pitch size revealed that the pitch size—badminton (6.1 × 13.4 m), volleyball (9 × 18 m), and basketball (14.2 × 26.5 m) had no significant difference in enjoyment [72]. The study was conducted on 12 overweight boys aged 10.7 ± 1.2 years. The researchers also found that three a side SSG could engage them for almost 80% of the time with moderate-to-vigorous PA (⩾3 metabolic equivalents (METs). In addition, the mean %HR_max_ was reported to range between 76 to 82%; and the majority of game time was spent at heart rate > 70% of HR_max_. There was no effect in ratings of perceived exertion (RPE) and enjoyment. Based on these findings it may be preferable to play SSG on a larger court when space is available. In general, it appears that SSG exercise intensity is increased with the concurrent reduction in player number and increase in relative pitch area per player [73]. The 30–40 min session was broken down into 5 min of warm up (stretching and football-specific warmups), 20–30 min of basic skills (kicking and dribbling), games (football, handball, and fun games), and 5 min of cool down. The duration of SSG fitted the standard school physical education. SSG was different from the standard school physical education (PE) lesson which emphasized physical and mental development and basic skills in sports. For example, for Standard 3 children (aged 9 years old), the focus included to master basic skill of movement in games and sports, to introduce health fitness concepts and to observe safety during exercise or sports activities [74]. Another feature of SSG is that it is a team sport. Previous study has shown that team sports are more fun and less likely to report anxiety and depression [36].

Each session was conducted by trained personnel (research assistant). The main sport was football; however, the trainers mix these games with handball and other games. Participants were advised to bring adequate drinking water to prevent dehydration. In the event of unforeseen circumstances such as bad weather, haze, or school event priorities, the sessions were changed to an indoor venue, postponed, or cancelled by the trainer or school authority. All trainers were provided first aid kits for emergency use. Furthermore, qualified sports medicine doctors and pediatricians were also appointed to monitor the health of the participants and were contactable throughout the study to offer medical consultation or treatment when necessary.

### Nutrition component

Nutrition education intervention (NEI) was specifically designed to address childhood obesity utilising nutrition education modules (NEM). This NEM was adapted from the Malaysian Childhood Obesity Treatment Trial (MASCOT)'s modules [75]. Unlike MyBFF@school, MASCOT study design was not a cluster randomised control trial. The investigators only adapted the relevant nutrition module from the MASCOT components. Modification had been made since MyBFF was a school based intervention and not a clinic based intervention as in MASCOT. A similar NEM was used for primary and secondary schoolchildren. The topics and aims of each topic are as shown in Table 1. Apart from classroom lectures, hands-on practical sessions or interactive activities were carried out to strengthen nutrition knowledge, attitude, and practices. NEI module was conducted at alternate week with a psychology component for 24 weeks by trained research assistants during the period of co-curriculum activities with 45–60 min per session.

**Table 1:** Topics and aims of the nutrition education intervention module

Topic	Aim
1. Wakeup call/Time to act Unit 1: Are You at Risk Unit 2: Challenges In Body Weight Loss And Management Unit 3: Time To Act	• Provide awareness to target groups (parents, teachers, and students) regarding unhealthy food intake practices • Give exposure to target groups (parents, canteen operators, teachers, and students) related to challenges in weight reduction and management • Inform parents regarding the nutritional intervention session that students will participate in the MyBFF@school program
2. My body weight/know my body weight	• Perform anthropometric measurements (height and weight) • Calculate body mass index (BMI) and plot BMI data on growth charts • Interpret the BMI z-score
3. Eat well, be well Unit 1: A Balancing Act / Count To Be Fit Unit 2: Fill In My Plate / Healthy Eating Plan Unit 3: Awesome Fruits And Veggie Unit 4: Plain Water is My Best Friend Unit 5: Less Salt And Fat, Snack Attack	• Knowing the concept of energy balance • Knowing the sources and amount of energy intake • Knowing the amount of energy consumption • Understanding the concept of the Malaysian Food Pyramid • Understand menu planning according to the Malaysia Healthy Plate • Explain the importance and how to increase intake of eating fruits and vegetables daily • Explain ways to increase the intake of fruits and vegetables • Understand the recommended amount and serving size of food • Describe one serving of fruits and vegetables for each meal • Know the importance of drinking plain water • Give guidance about the selection of low-salt and low-fat foods as well as healthy snacks
4. Make a better life Unit 1: Breakfast Power	• The importance of breakfast • Benefits of breakfast to learning • Healthy breakfast options
5. My Body is Fit and Fabulous (MyBFF) Unit 1: Smart Shopping Unit 2: Let’s Cook / Prepare Meal Together Unit 3: Eating Out: Fast Food / Food Outlet / School Canteen / Hawkers	• Guide students in how to read and understand nutrition labels and choose food wisely • Guide students in how healthy food/meals are provided for families • Guide students in how to choose healthy food options when eating out

### Psychology component

The psychology module was developed to strengthen the psychological aspects of the schoolchildren and prevent dropout from the program. The content of the module was arranged based on literature review and psychological theories [76] with its objective being to help in fostering schoolchildren’s willpower in sustaining their participation in the program toward the end. This module consisted of five psychological aspects, including activities to be carried out by the children. The main themes in the modules were self-esteem, friendship, assertiveness, and positive thinking for a healthy lifestyle and stress management. Obese children generally need to be strengthened in self-esteem, friendship, assertive, positive thinking for a healthy lifestyle, and stress management in order for them to be able to sustain the intervention program and have confidence in reducing their body weight. This results from the fact that they usually face stigmatization [77]. However, all five aspects were found to produce positive impacts on HRQOL and psychological well-being [78, 79]. It also aimed to raise schoolchildren’s confidence in the ability of the program to help them reduce weight, thereby enjoying a healthy lifestyle. The units of this module were carefully designed to build resiliency among the schoolchildren, particularly in helping them overcome challenges due to stigmatization. The main themes in the modules were self-esteem, friendship, assertiveness, and positive thinking for a healthy lifestyle and stress management. Psychology sessions were conducted twice a month, with 30–45 min allocated per session. The participants were gathered in the hall or classroom and were divided into smaller groups. Each group was guided by trained personnel (research assistant). Motivational talks and interactive activities were the two main mechanisms used to achieve the objectives of the psychology module during these sessions. The activities were carried out both indoors and outdoors. At the end of the activities, a reflection session was conducted for the schoolchildren to review their opinion on the activities.

### Comparison to the existing program for the control

The control schools followed the existing standard program i.e. the Malaysian national curriculum i.e. differing from MyBFF@school by the health education and physical education sessions. Health education is incorporated in the standard national curriculum for primary (age 7–12 years) and secondary school (aged 13–17 years) and is mandatory for all government schools. The standard contents are nutrition, mental health, personal health including personal hygiene, reproductive health, and selected diseases [80, 81]. The content also has safety and first-aid. This health education subject is taught over 35 sessions per year with each session lasting for 30–40 min (primary: 30 min, secondary: 40 min). However, the nutrition component is only allocated five sessions (15%). In addition, the standard health education lacks practical or interactive sessions in the nutrition component. Psychology topics in health education include emotional well-being and conflict and stress management. MyBFF@school’s psychology module covers more scope e.g. self-esteem, friendship, assertiveness, and positive thinking for a healthy lifestyle and stress management. Meanwhile, physical education sessions last for 30–40 min and the standard content is based on developing basic movement and game skills and emphasizes on correct technique. From our observation, there is less game play as compared to SSG. The sports in the syllabus included several popular games including football, netball, racquet sports, and track and field. Following our discussion with the stakeholders, especially the Educational Planning and Research Division (EPRD), Ministry of Education Malaysia, allowed us to replace PE sessions with MyBFF@school’s SSG. However, the health education subject is not replaced, as this topic has exams and carries marks for the schoolchildren. MyBFF@school’s nutrition and psychology modules were allowed to replace the standard co-curricular sessions in the intervention schools. The standard co-curricular sessions were typically conducted once a week, one hour per session, within the months of February until October of the school academic year.

### Training for MyBFF@school personnel

All personnel (trainers/research assistants) were graduates with a basic degree in nutrition (majority), allied health sciences (physiotherapy), and sports science. They underwent two structured training programs, including physical activity, nutrition, and psychology modules for a duration of four days for each program. All training was conducted by the research team who are sports physicians, sports (football) coach, nutritionists and psychology experts (PhD in psychology). A certificate of competency was issued upon completion of training. Two further refresher courses were conducted during the intervention to consolidate skills.

### Assessment and follow-up

Socio-demographic data, anthropometric measurements, clinical assessments, and interviewer-assisted questionnaires were conducted at baseline, after three months and at the end-point after six months. Body composition, clinical parameters and fitness test were assessed at baseline, three and six months. Nutrition and psychology components as well as blood parameters were assessed only at baseline and six months. Prior to the study visit, the participants were asked to fast overnight for at least eight hours. All anthropometric measurements were performed by trained personnel, and health examinations were performed by pediatricians.

### Anthropometric measurement

Standing height was measured to the nearest 0.1 cm using a calibrated stadiometer (Seca 217, Germany), with the participants not wearing shoes. Body weight and body composition were measured to the nearest 0.1 kg using a pre-calibrated body impedance analyzer (InBody 720, Korea), with the participants in light clothing and without shoes and socks. WHO AnthroPlus 2007 software was used to calculate BMI $z$-score. According to the WHO BMI chart (2008), overweight, obese, and morbidly obese were defined by BMI $z$-scores more than 1, 2, and 3 SDs, respectively, adjusted for age and gender [82]. Using a non-extensible tape (Seca 201, Germany), waist circumference was measured twice to the nearest 0.1 cm over the skin midway between the 10th rib and the iliac crest after normal expiration, and the mean was recorded.

### Clinical measurement

Two readings of blood pressure were measured after 5 min of resting using a mercury sphygmomanometer (Accoson, UK). Measurements were taken with the participants in a seated position and with the arm supported at the heart level, and the mean was recorded. Pubertal status was assessed (self-administered) using Tanner staging scale, and participants were examined for the presence of acanthosis nigricans over the neck.

Venipuncture was performed by experienced nurses and doctors. Approximately 15 mL of fasting, venous blood was taken from the participants for standard biochemical tests to determine glucose, HbA1c, lipid profiles, liver enzymes, and fasting insulin. Blood samples were transported cold at 4–8 °C in iceboxes to the central laboratory at the Institute for Medical Research within two hours of collection and processed on the same day. Consent was obtained from the parents or guardians of participants for permission to store remaining blood samples for future biomarker research related to obesity. Participants with abnormal blood results were referred to the nearest clinic or hospital for further examination and management.

### Physical fitness measurement

Fitness tests were conducted using a modified Harvard step test. Participants were screened prior to the test to exclude individuals with pre-existing medical conditions or musculoskeletal injuries. The modified Harvard step test is a convenient modality for predicting aerobic fitness by measuring post-exercise recovery heart rates in children [83], and is easily administered since it requires minimal portal equipment and has a short duration. Stepping skills require little practice and can be mass tested. This approach is currently being used by schoolteachers as the standardized protocol for fitness assessment in all schoolchildren. The test has been shown to be moderately reliable with intraclass correlation coefficient of 0.62 and is recommended from other aerobic (cardiorespiratory) fitness tests to be used in sports and occupational settings [84].

### Nutrition knowledge, attitude and practice measurement

A similar pretested nutrition module questionnaire was used both at baseline and post-intervention (after six months). It was administered by the same research team for both primary and secondary schoolchildren in the intervention and control groups to assess their nutrition knowledge, attitude and practices (KAP). Both the nutrition knowledge and attitude questionnaire for the primary and secondary school children were pre-tested and the internal consistency was also checked. For the primary school, the reliability of 10-question on the general nutrition knowledge was Cronbach’s alpha coefficient of 0.673, while the 15-question key nutrition attitude component was with Cronbach’s alpha coefficient of 0.679. As for the secondary school, the Cronbach's alpha coefficient for the 10-item nutrition knowledge scale was 0.537 while nutrition attitude assessment which consisted of 15 nutrition attitude questions was 0.657.

As for the nutrition practice, no reliability and validity test was conducted. Thus, the nutrition practice was measured using quantitative food frequency questionnaire (FFQ) for the past one week which was adopted for FFQ on fruit and vegetable from the WHO STEPwise Approach for Surveillance of Non-Communicable Diseases [85] and this similar approach was previously used in the NHMS 2011 and NHMS 2015 in Malaysia. Apart from that, questionnaires on the Trans-theoretical Model (TTM) [86] were only directed for the secondary schoolchildren to assess their stages of change on weight management as well as fruit and vegetable intake.

### Psychology well-being measurement

Data for the psychological aspects have been collected via different instruments for primary and secondary schools. All approaches used in the study were selected based on the desired area of assessment of the study. Questionnaires were translated from English to the local language, back-translated, pretested, and revised accordingly. After translation, the questionnaires were piloted in schools in Putrajaya in 2015. All were found to have a Cronbach’s alpha value exceeding 0.70 during the pilot study.

As for primary school, KINDL^R^, Stirling Children’s Well-being Scale (SCWBS), and Children’s Eating Attitude Test (ChEAT) have been used to gather data from the samples. KINDL^R^ is a self-report questionnaire with 24 items to measure health-related quality of life (HRQOL) using six dimensions, namely, physical well-being, emotional well-being, self-esteem, family, friends, and school [87, 88]. 5-point Likert scale is used to rate all the items. SCWBS consists of 12 items that measure emotional and psychological wellbeing in children aged 8 to 15 years old. All items are rated on a 5-point Likert scale. Three sub-scales in SCWBS are positive emotional state, positive outlook and social desirability [89]. ChEAT is a self-reported questionnaire aims to measure eating behavior and attitudes of the children [90]. ChEAT comprises of 26 items which are rated on a 6-point Likert scale with responses such as always, very often, often, sometimes, rarely and never. The subscales for ChEAT are body/weight concern, dieting, food preoccupation and eating concern [91].

Youth Self-Report (YSR) has been used to obtain psychological data for secondary schools. YSR is a child-report questionnaire which assess the problem behaviours along two broad scales namely internalizing and externalizing. 3-point Likert scale is used as responses to the 112 items in YSR. YSR has been developed based on seven subscales, namely activities, social, anxious/depressed, withdrawn/depressed, somatic complaints, social problems and thought problems [92].

## Data management and statistical analysis

Study data were collected and managed using Research Electronic Data Capture (REDCap) hosted at the University of Malaya. REDCap is a secure, web-based application designed to support data capture for research studies and provide an intuitive interface for validated data entry, auditing trials for tracking data manipulation and export procedures, automation of export procedures for seamless data downloads to common statistical packages, and managing procedures for importing data from external sources. Quality control was performed by randomly comparing 10% of the data from the database with the raw data in order to ensure its consistency. All analyses were conducted at 5% significant level using STATA Version 14 and SPSS IBM version 24.

### Descriptive statistics

Sociodemographic information, anthropometric measurement and factors related to the outcomes of interest were produced. Categorical data was presented as frequency and percentage. Distribution for numerical data were tested. Mean and standard deviation were presented for normally distributed data. While median and interquartile range for non-normally distributed data. The association between categorical variables was measured using Chi-square statistics. For numerical data, appropriate statistical analysis depending on the type of distribution and number of comparison groups.

### Cross-sectional analysis

At baseline, the association between outcomes of interest and exposures with its covariates were measured by linear regression or logistic regression where appropriate. Model building strategies involving testing the univariate associations. Consequently, covariates with a* p*-value of less than 0.25 were included in the multivariable regression models. Covariates with clinical significance were considered in the multivariate regression models. Goodness of fit for the final linear regression model is determined based on R-squared. While, the goodness of fit for the final logistic regression model were tested using Hosmer–Lemeshow goodness of fit test, classification table and ROC curve.

### Assessment of treatment effect

The effect of the intervention will be assessed using intention-to-treat basis. Changes of outcomes on the continuous scale from baseline to the end of follow-up between intervention and control will be assessed using the mixed effect model accounting the cluster effect. Analysis of categorical outcome will be done using Generalized Estimating Equation (GEE), controlling for gender and other potential confounding factors.

### Missing data management

Prior to longitudinal data analysis, the completeness of data was checked. The missingness of data with missing values with more than 5% were determined either missing completely at random (MCAR), missing at random (MAR) or missing not at random (MNAR) where appropriate. MAR or MNAR data were imputed using multiple imputation (MI).

## Results

**Fig. 1:**
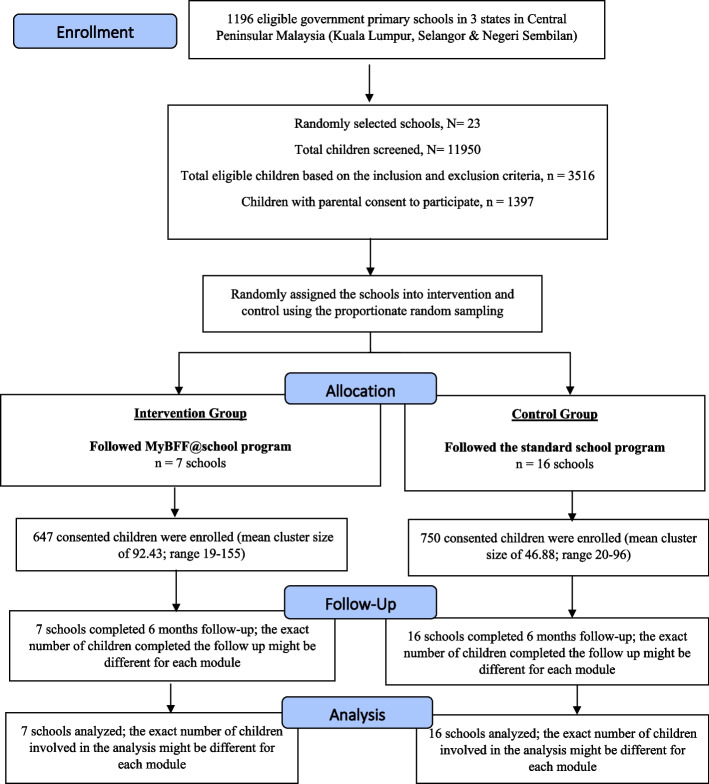
CONSORT diagram of MyBFF@school intervention program in primary school

**Fig. 2:**
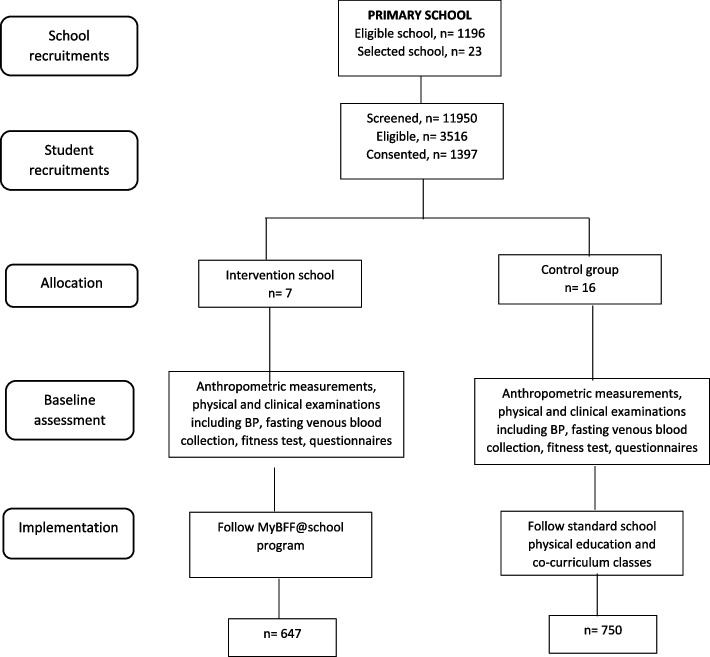
Flowchart of MyBFF@school program in primary school

**Fig. 3:**
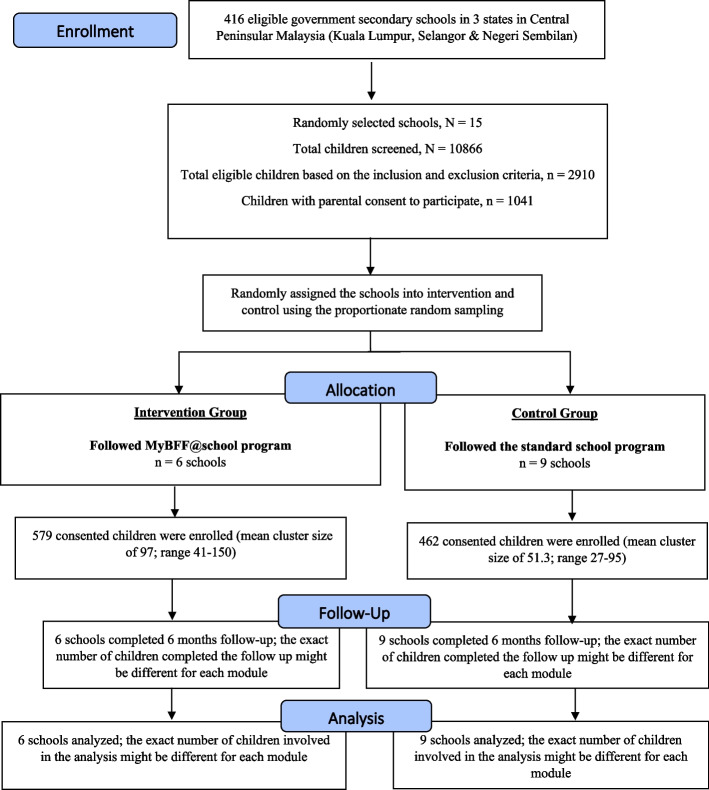
CONSORT diagram of MyBFF@school intervention program in secondary school

**Fig. 4:**
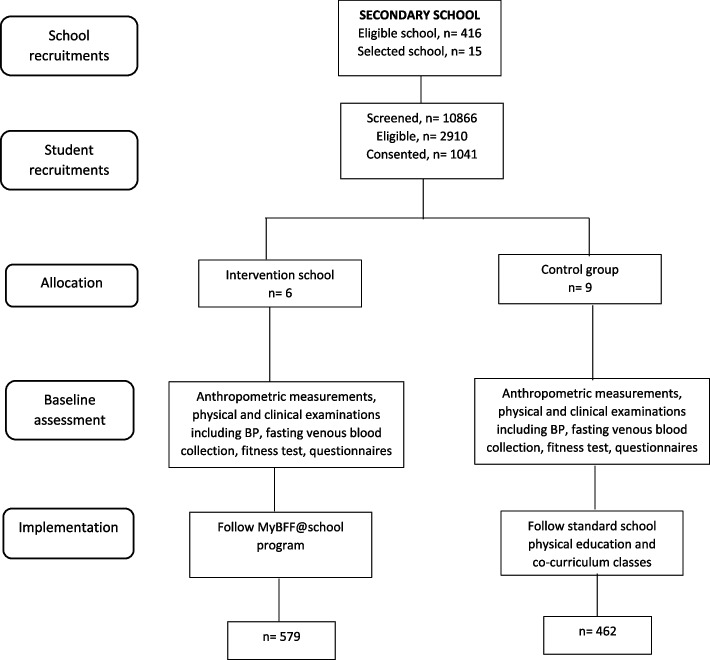
Flowchart of MyBFF@school program in secondary school

As shown in Figs. 1 and 2, there were 23 primary schools participated in the study randomly allocated into intervention (seven school; 647 consented children; mean cluster size of 92.43; range 19–155) and control (16 schools; 750 consented children; mean cluster size of 46.88; range 20–96). As for the secondary school (Figs. 3 and 4), a total of 15 secondary schools participated which randomly assigned into intervention (6 schools; 579 consented children; mean cluster size of 97; range 41–150) and control (9 schools; 462 consented children; mean cluster size of 51.3; range 27–95). From the pre-screening conducted before beginning this study, the prevalence of overweight and obese schoolchildren in primary school was found to be 29.4%, whereas the prevalence in secondary schoolchildren was slightly less at 26.8%. The participation rates of the schoolchildren for this study were 39.7% and 35.8% for primary and secondary, respectively. Initially, there were 1,397 participants at baseline for primary schoolchildren. At month-3 visit, 1,200 (85.9%) completed the assessment and at month-6 visit 1,113 (79.7%) completed the assessment. However, only 995 (71.2%) participants completed all three visits. For secondary schoolchildren, 1,041 participants completed baseline assessment, followed by 787 (75.6%) at month-3 and 730 (70.1%) at month-6 visits. Similarly, a lesser number of them completed all three visits i.e. 619 (59.5%).

The baseline characteristics of the primary and secondary schools’ participants are summarized in Table 2. A total of 1,397 primary schoolchildren aged 9–11 and a total of 1,041 secondary schoolchildren aged 13–16 were enrolled in the MyBFF@school program. The mean age did not differ between boys and girls within primary and secondary school participants (9.9 versus 9.8 years and 14.1 versus 14.2 years respectively, $p>0.05$). Among primary school participants, boys had significantly higher BMI *z*-score (2.45 versus 2.15), and waist circumference (75.7 cm versus 74.2 cm), but significantly lower in percentage body fat (37.2% versus 38.5%) and abdominal obesity (56.8% versus 78.6%) when compared to girls (all $p<0.001$). Skeletal muscle mass did not differ between boys and girls in primary school. Similarly, boys in secondary school had significantly higher BMI *z*-score (2.23 versus 2.07), waist circumference (88.9 cm versus 83.2 cm) and skeletal muscle mass (25.9 kg versus 21.1 kg), but lower in percentage body fat (35.5% versus 42.0%) when compared to girls (all $p<0.001$). However, abdominal obesity did not differ between boys and girls in secondary school. In addition, Table 2 also shows the distribution of obesity status among primary and secondary schools’ participants. Among primary school participants, 39.7% were overweight, 42.7% were obese and 17.6% were morbidly obese. Among secondary school participants, 46.6% were overweight, 42.0% were obese, and 11.4% were morbidly obese. Finally, the distribution of overweight, obese and morbidly obese significantly differed between boys and girls in both primary and secondary school participants (all $p<0.001$).

**Table 2:** Baseline anthropometric and demographic parameters among MyBFF@school participants in primary and secondary schools. Means at baseline were compared between boys and the girls using independent samples *t*-tests. Baseline categorical variables were compared using the chi-squared test

Characteristics	Primary school				Secondary school			
	**(n = 1397)**				**(n = 1041)**			
	**Overall**	**Boys**	**Girls**	***p*-value**	**Overall**	**Boys**	**Girls**	***p*-value**
**Age (years), mean (SD)**	9.9 (0.8)	9.9 (0.8)	9.8 (0.8)	NS	14.2 (1.4)	14.1 (1.4)	14.2 (1.3)	NS
**Gender, n (%):**								
Boys	751 (53.8)				430 (41.3)			
Girls	646 (46.2)				611 (58.7)			
**Ethnicity, n (%):**								
Malay	821 (58.8)	413 (66.4)	408 (73.5)	< 0.001	700 (67.2)	282 (75.2)	418 (75.5)	0.03
Chinese	214 (15.3)	139 (22.3)	75 (13.5)		58 (5.6)	16 (4.3)	42 (7.6)	
Indian	97 (6.9)	44 (7.1)	53 (9.5)		136 (13.1)	66 (17.6)	70 (12.6)	
Others	45 (3.2)	26 (4.2)	19 (3.4)		35 (3.4)	11 (2.9)	24 (4.3)	
**Location, n (%):**								
Urban	865 (61.9)	472 (62.8)	393 (60.8)	NS	666 (64.0)	288 (67.0)	378 (61.9)	NS
Rural	532 (38.1)	279 (37.2)	253 (39.2)		375 (36.0)	142 (33.0)	233 (38.1)	
**BMI *z*-score, mean (SD)**	+ 2.29 (0.81)	+ 2.42 (0.86)	+ 2.15 (0.73)	< 0.001	+ 2.10 (0.71)	+ 2.23 (0.72)	+ 2.07 (0.72)	< 0.001
**Waist circumference (cm), mean (SD)**	75.0 (9.6)	75.7 (9.8)	74.2 (9.2)	< 0.001	85.5 (10.9)	88.9 (11.2)	83.2 (10.1)	< 0.001
**Percentage body fat (%), mean (SD)**	37.8 (6.5)	37.2 (6.8)	38.5 (6.2)	< 0.001	39.2 (7.3)	35.5 (7.9)	42.0 (5.2)	< 0.001
**Skeletal muscle mass (kg), mean (SD)**	14.7 (2.9)	14.7 (2.7)	14.6 (3.2)	NS	23.1 (5.2)	25.9 (6.0)	21.1 (3.3)	< 0.001
**Weight status, n (%):**								
Overweight (BMI *z*-score > + 1 and ≤ + 2)	554 (39.7)	261 (34.8)	292 (45.2)	< 0.001	485 (46.6)	164 (38.1)	321 (52.5)	< 0.001
Obese (BMI *z*-score > + 2 and ≤ + 3)	597 (42.7)	320 (42.6)	277 (42.9)		437 (42.0)	211 (49.1)	226 (37.0)	
Morbidly obese (BMI *z*-score > + 3)	246 (17.6)	170 (22.6)	77 (11.9)		119 (11.4)	55 (12.8)	64 (10.5)	
**Abdominal obesity** (waist circumference ≥ 90th percentile)	926 (66.9)	424 (56.8)	502 (78.6)	< 0.001	644 (62.0)	263 (61.2)	381 (62.7)	NS

*SD* Standard deviation, *BMI* Body mass index, *NS* Not significant

## Discussion

The MyBFF@school program is a multi-faceted intervention program comprising physical activity, nutritional and psychological components to enhance the study outcome. One of the distinctive aspects of this study is the implementation of physical activity in the form of small-sided games i.e. SSG. SSG was adapted from small-sided games used by team sports in their training [93]. Most previous childhood obesity interventions did not apply physical activity intervention in the form of SSG [5, 94–97]. A module close to the SSG was used in a small study in obese children, but in the form of recreational soccer program [98] , and futsal [99]. SSG were designed in a way that encourages more interaction, involvement, and movement of the children in order to maximize their physical activity level when playing the games. A recent study reported higher heart rates in children playing soccer (aged 12–16 years) in the form of SSGs compared to large-sided games [100]. Furthermore, other studies have reported that SSG have similar effects to high-intensity interval training on fitness and endurance of youth soccer players (mean age 16.2 ± 1.6 years) [101].

The overweight and obese children participating in this study were isolated from their peers in order to be approached as a target group and directly address their excess weight issues. Although there is fear of stigmatization from their peers, it is imperative to apply this approach as overweight and obese children might not be able to fully engage in the intervention, particularly when playing SSGs, in which they have a tendency to be withdrawn or lag behind their non-overweight and non-obese peers. Furthermore, by isolating these schoolchildren, the investigators were able to address issues of self-stigma through dedicated psychology session, since their weight-based self-stigma was higher than that in children of normal weight [102]. Overweight schoolchildren are faced with many psychological barriers due to stigma [94], and they were thus grouped together with peers of similar weight to help in forming a support group for sustaining their participation in the intervention and reducing the effect of the stigma. Forming a support group composed of children with similar weights is advantageous, since studies reveal that children tend to more easily form friendships with children of similar BMI [103].

Morbidly obese was included in the weight category as the investigators predicted that it would influence the outcome. Although differences in the physical activity levels of normal weight, overweight, and obese children have been reported [104], morbidly obese individual may behave differently. For example, morbidly obese women have been found to perceive significantly more discomfort and pain in a 6 min walking test compared to obese and lean women [105]. Owing to differing perceptions of discomfort and pain in physical activities, the investigators predicted that the three categories will produce different results. Additionally, a previous study found a correlation between BMI and PBF among children [106]. A surgical intervention, for example, a bariatric bypass using stomach stapling, has been predominantly reserved for the morbidly obese [107, 108]. Hence, it is important for us to consider the severity of obesity as this may be helpful in both planning the intervention and explaining its results.

In addition, the MyBFF@school with nutrition education intervention component had included topics that were different from a typical national school syllabus for example, the “Let’s Cook” and “Smart Shopping” ideas that help in empowering children to make healthier choices. The global nutrition transition is caused by the availability of cheap edible oil and processed foods that usually contain high amounts of fat, salt, and added sugar. Consequently, global trends toward increased usage of caloric sweeteners, increased intake of animal-source food, and decreased intake of legumes, coarse grains, and vegetables have occurred during the recent decades [109]. This transition and shift in diets contributed to the obesity pandemic, and they make healthy food choices increasingly important. It is thus crucial to enrich children with nutritional knowledge in order to increase their understanding of the benefits of healthy dietary practices and foster a preference for healthy foods. Theoretically, increasing awareness about healthy nutrition-related habits could stimulate changes toward more healthy dietary practices [110]. The nutrition education module in MyBFF@school program provides both practical knowledge and experience to drive changes in nutrition-related practice. The unit called “Smart Shopping” gives the children practical experience in selecting healthy food and in reading and understanding food labels. Likewise, the children benefited from the unit termed “Let’s Cook” by gaining hands-on experience in preparing healthy balanced meals. The module was also packed with a multitude of interactive activities to provide an effective platform for the children to learn by practice [111].

Childhood obesity intervention programs usually aim to reduce percentage body fat by focusing on nutrition and physical activity without directly addressing the well-being of the children concerned [112–114]. In light of the aforementioned issues, the MyBFF@school program incorporated a significant psychological aspect. This is supported by a longitudinal study by Shoshani et al. (2014) that found that positive psychology intervention could enhance the well-being of school-aged children [115].

Hence, the investigators included psychology aspects in the intervention, which is remarkable compared to previous studies. Obese children often face weight-based stigma that harms their psychological well-being [115–117]. Moreover, overweight children are highly likely to develop self-stigma related to a lower level of HRQOL [118]. Faced with weight-based stigma, these children could be trapped in a maladaptive cycle whereby weight stigma induces weight gain because of stressful experiences [119]. This stigma could also worsen their weight problem, since induced behavior such as binge eating and social isolation may reduce their quality of life [120]. Additionally, overweight and obese children are disproportionately involved in bullying, either as the victim or the perpetrator [121, 122]. This could affect their psychological well-being; indeed, children involved in bullying are more likely to report worse symptoms of depression [123].

Overweight and obese children are more likely to experience psychosocial problems, such as a low quality of life, impaired social functioning, low self-esteem, depression, and eating disorders [124]. A study by Schwimmer et al., revealed that the level of HRQOL among obese children is similar to children diagnosed with cancer [125]. Being overweight and obese are also associated with body dissatisfaction and low self-esteem that could bring a detrimental effect to emotional and psychological well-being [117, 126–128]. Thus, early, intervention is crucial to curb issues of declining emotional and psychological well-being in overweight and obese children.

The prevalence of overweight and obese children found in this study was similar to the national and global prevalence of overweight and obese children. Of 22,816 children screened, the investigators found high prevalence of overweight and obesity i.e. 29.4% and 26.8% among 9–11 year-old and 13–16 year-old children respectively. This is consistent with the Malaysian NHMS 2019 data, which found that the national prevalence of overweight and obese children aged 10–17 years was 29.8% (26.2% of girls and 33.2% of boys) [10]. Globally, 24% of girls and 27% of boys aged 5–19 years were overweight and obese according to the WHO 2016 report [129]. Generally, it seems that the prevalence rates of overweight and obese children in Malaysia are both higher than those in the developing countries and the WHO figure [130, 131]. This further reflects the need for an effective intervention in the Malaysian population.

The MyBFF@school program was conducted during school hours replacing the periods of Physical Education and co-curriculum. Full-time trained personnel were stationed at each school to ensure delivery of the intervention package to the schoolchildren and to enable good rapport among the schoolchildren, teachers, and parents and ensure their commitment to this study. The research assistants were monitored by a central/field supervisor. Contamination effects were reduced by taking into account, for example, the distance between the schools in the intervention group and those in the control group. Intervention was carried out in schools rather than in isolated lab settings to ensure that the children were always in the natural environment throughout the study. The children were naturally exposed to a multitude of external factors outside of school that could also affect the outcome of this study. Thus, if a significant effect is observed in this study, the intervention is shown to be feasible for implementation at school level.

The mean BMI* z*-score for boys was significantly higher than that for girls in both primary and secondary schoolchildren, indicating differences in gender. The waist circumferences of boys in primary and secondary schools are substantially higher than those of girls. On the contrary, percentage body fat (PBF) is higher in girls than in boys. Previous studies suggested that girls aged 3–8 years had approximately 50% more body fat compared to boys of the same age, weight, and height [132]. Furthermore, the mean skeletal muscle mass in boys is significantly higher than that in girls for both primary and secondary schools.

MyBFF@school program has several advantages compared to other intervention studies. One of them includes the fact that this study was conducted during school hours, and the MyBFF@school program was incorporated into the existing national curriculum and integrated as a co-curriculum activity. Full-time trained personnel were stationed at each school to ensure delivery of the intervention package to the schoolchildren and to enable good rapport among the schoolchildren, teachers, and parents and ensure their commitment to this study. The research assistants were monitored by a central/field supervisor. Contamination effects were reduced by taking into account, for example, the distance between the schools in the intervention group and those in the control group. Intervention was carried out in schools rather than in isolated lab settings to ensure that the children were always in a common environment throughout the study. The children were naturally exposed to a multitude of external factors outside of school that could also affect the outcome of this study. Thus, if a significant effect is observed in this study, the intervention is shown to be feasible for implementation at school level.

However, there were a few limitations that the investigators need to address. One of the limitations of this study is the lack of control outside of school because of the absence of direct parental involvement in the intervention program. Much research has noted the importance of parental involvement in school activities [133]. Unfortunately, this was beyond the scope of the study. An information bias in the study could also exist because of the application of self-administered questionnaires during data collection. In particular, questionnaires regarding socio-demographic characteristics were not completed by all parents, and such information could not be inferred from the participants alone. Despite attempts via telephone calls to obtain this information, the investigators did not receive full cooperation to overcome this issue. Another limitation was total uniformity of program delivery across the experimental schools could not be ensured. However, the investigators tried to improve the limitation by the researchers conducting interval visits (twice) to the sites besides the refresher courses that were conducted for the trainers during school breaks.

## Conclusion

MyBFF@school screening prior to the intervention found a high prevalence of overweight and obesity among schoolchildren. The prevalence of overweight and obesity was high; 29.4% among the primary schoolchildren and 26.8% in secondary schoolchildren. This resonates the need for an urgent and effective school-based intervention program in the country. The MyBFF@school intervention, a multi-component program consisting of physical activity, nutrition, and psychology was designed taking into consideration the feasibility, viability and practicality to combat this problem.

## Data Availability

All relevant data are within the paper.

## Abbreviations

ANCOVA: Analysis of covariance
ANOVA: Analysis of variance
BMI: Body mass index
ChEAT: Children’s Eating Attitude Test
C-RCT: Cluster Randomized Controlled Trial
FFQ: Food Frequency Questionnaire
GEE: Generalized estimating equation
HbA1c: Hemoglobin A1c
HRQOL: Health-related quality of life
ICC: Intraclass correlation coefficient
KAP: Knowledge, attitude, practices
MAR: Missing at random
MASCOT: Malaysian Childhood Obesity Treatment Trial
MCAR: Missing completely at random
MEM: Mixed-effect linear model
MET: Metabolic equivalent
MI: Multiple imputation
MNAR: Missing not at random
MVPA: Moderate-to-vigorous physical activity
NCD: Non-communicable disease
NEI: Nutrition Education Intervention
NEM: Nutrition Education Modules
NHMS: National Health and Morbidity Survey
PA: Physical activity
PBF: Percentage body fat
PE: Physical education
PT3: Form Three Assessment
REDCap: Research Electronic Data Capture
ROC: Receiver operating characteristic
RPE: Ratings of perceived exertion
SCWBS: Stirling Children’s Well-being Scale
SD: Standard deviation
SSG: Small-sided games
TTM: Trans-theoretical Model
UPSR: Primary School Achievement Test
WHO: World Health Organization
